# Specific killing of DNA damage-response deficient cells with inhibitors of poly(ADP-ribose) glycohydrolase

**DOI:** 10.1016/j.dnarep.2017.02.010

**Published:** 2017-04

**Authors:** Polly Gravells, Emma Grant, Kate M. Smith, Dominic I. James, Helen E. Bryant

**Affiliations:** a Academic Unit of Molecular Oncology, Sheffield Institute for Nucleic Acids (SInFoNiA), Department of Oncology and Metabolism, University of Sheffield, Beech Hill Road, Sheffield, S10 2RX, United Kingdom; b Drug Discovery Unit, Cancer Research UK Manchester Institute, The University of Manchester, Wilmslow Road, Manchester, M20 4BX, United Kingdom

**Keywords:** Synthetic lethality, Breast cancer, PARG inhibition, FAM175A (ABRAXAS), BRCA1, PALB2, BARD1

## Abstract

Poly(ADP-ribosylation) of proteins following DNA damage is well studied and the use of poly(ADP-ribose) polymerase (PARP) inhibitors as therapeutic agents is an exciting prospect for the treatment of many cancers. Poly(ADP-ribose) glycohydrolase (PARG) has endo- and exoglycosidase activities which can cleave glycosidic bonds, rapidly reversing the action of PARP enzymes. Like addition of poly(ADP-ribose) (PAR) by PARP, removal of PAR by PARG is also thought to be required for repair of DNA strand breaks and for continued replication at perturbed forks. Here we use siRNA to show a synthetic lethal relationship between PARG and BRCA1, BRCA2, PALB2, FAM175A (ABRAXAS) and BARD1. In addition, we demonstrate that MCF7 cells depleted of these proteins are sensitive to Gallotannin and a novel and specific PARG inhibitor PDD00017273. We confirm that PARG inhibition increases endogenous DNA damage, stalls replication forks and increases homologous recombination, and propose that it is the lack of homologous recombination (HR) proteins at PARG inhibitor-induced stalled replication forks that induces cell death. Interestingly not all genes that are synthetically lethal with PARP result in sensitivity to PARG inhibitors, suggesting that although there is overlap, the functions of PARP and PARG may not be completely identical. These data together add further evidence to the possibility that single treatment therapy with PARG inhibitors could be used for treatment of certain HR deficient tumours and provide insight into the relationship between PARP, PARG and the processes of DNA repair.

## Highlights

• A synthetic lethal screen for Poly(ADP-ribose)glycohydrolase (PARG) is presented.
• SiRNA and the PARG inhibitors Gallotannin and PDD00017273 are used.
• PARG is synthetically lethal with BRCA1, BRCA2, PALB2, FAM175A (ABRAXAS) and BARD1.
• PARG inhibition induces DNA damage, stalled replication forks and homologous recombination.
• The data support the validity of PARG as a target for therapy.

## 1 Introduction

An early response to DNA damage is the addition of poly(ADP-ribose) (PAR) to proteins [1], [2]. This post-translational modification has been implicated in repair of single [3], [4], [5], [6], [7] and double strand DNA breaks [8], [9], [10], [11] and in the restart of stalled or collapsed DNA replication forks [12], [13], [14]. In each case, poly(ADP-ribose) polymerase (PARP) enzymes are recruited to and activated at sites of damage. Using NAD as a substrate, they add multiple ADP-ribose subunits to gamma carboxyl groups of the glutamic acid residues of acceptor proteins [15]. The PAR signal is then assumed to recruit other repair factors to the site of damage allowing DNA repair and/or continued DNA replication [11], [14], [16], [17], [18].

We and others demonstrated that PARP inhibitors can specifically kill homologous recombination repair (HRR) deficient tumours [19], [20]. This is because, in the absence of PARP activity, increased numbers of replication forks collapse and HRR becomes essential for cell survival [14], [19], [21]. Accordingly, inhibition of PARP has become a successful and increasingly promising therapeutic approach for certain tumour types [22].

Nevertheless, PAR modification is reversible and it is proposed that once other repair proteins have localized to the damaged DNA, PAR needs to be removed before repair can take place [6]. Poly(ADP-ribose) glycohydrolase (PARG) has -endo- and exoglycosidase activities which can cleave glycosidic bonds, rapidly reversing the action of PARP enzymes and returning proteins to their native unmodified state [23], [24], [25], [26], [27]. Like PARP, PARG is rapidly recruited to sites of DNA damage, and cells deficient in the nuclear form of PARG display increased levels of genomic instability and are sensitive to DNA damaging agents [28], [29], [30], [31], [32], [33], [34]. Consistent with an essential role in reversing PARP activity at sites of DNA damage, PARG activity is reported to contribute to the efficiency of DSB and SSB repair, to be required for recovery from prolonged replication stress and to have a role in resolving unusual replication structures in S-phase [6], [35], [36], [37], [38]. We previously demonstrated a synthetic lethal relationship between PARG and the HRR protein BRCA2, which was suggested to be due to increased collapsed replication forks that could not be resolved by HRR [39]. Here we screen for other genetic alterations which maybe synthetic lethal with PARG, and further investigate the mechanism by which this occurs.

We demonstrate that along with the previously shown BRCA2, BRCA1, PALB2, FAM175A (ABRAXAS) and BARD1 are all synthetic lethal with siRNA mediated depletion of PARG and with inhibition of PARG enzyme activity. We confirm directly that PARG inhibition leads to stalled replication forks and increased HRR. Further, we demonstrate that each of the genes above is required for efficient HRR, suggesting that, as with BRCA2, it is the lack of HRR proteins at PARG inhibitor-induced replication stalling that induces cell death. These data together add further evidence to the possibility that single treatment therapy with PARG inhibitors could be used for treatment of HRR-deficient tumours.

## 2 Material and methods

### 2.1 Cell culture

The MCF7 breast epithelial adenocarcinoma cell line was purchased from the American Type Culture Collection (ATCC^®^ HTB-22™). This cell line was grown in Dulbecco’s modified Eagle Medium (DMEM) supplemented with 1x non-essential amino acids (NEAA, Sigma) and 10% Foetal bovine serum (Gibco) at 37 °C under an atmosphere containing 5% CO_2_.

### 2.2 Inhibitors

The PARG inhibitor PDD00017273 [40] was resuspended in DMSO at a concentration of 20 mM. Final concentrations were 0.3 μM and 1 μM unless otherwise stated. The commercially available PARG inhibitor, gallotannin (C_76_H_52_O_46_) (GLTN) was purchased from Enzo Life Sciences (ALX-270-418). 1000 × stocks were prepared in sterile H_2_O and stored at −20 °C. A final concentration of 10 μM was used. The PARP inhibitor, olaparib was purchased from Cambridge Biosciences and prepared in dimethyl sulfoxide (DMSO) to give a 1000 × stock. Unless otherwise stated, a final concentration of 1 μM was used.

### 2.3 SiRNA screen

A custom-made 96-well plate was purchased from Dharmacon (GE Healthcare) containing four individual ON-TARGETplus siRNA oligonucleotides for each of the following 18 genes; *BRCA1* (NM_007298), *BRCA2* (NM_000059), *PALB2* (NM_024675), *RAD51C* (NM_002876), *CHEK2* (NM_145862), *RAD51D* (NM_133629), *BRIP1* (NM_032043), *BARD1* (NM_000465), *MRE11A* (NM_005591), *NBN* (NM_002485), *RAD50* (NM_133482), *TP53* (NM_000546), *PTEN* (NM_000314), *STK11* (NM_000455), *ATM* (NM_138292), *FAM175A* (NM_139076), *XRCC2* (NM_005431) and *CDKN2A* (NM_058195). Each of the four were tested for efficiency of mRNA depletion and the two with greatest effect were taken forward into the screen. ON-TARGETplus siRNA was also purchased from Dharmacon for two individual PARG (NM_003631) siRNA oligonucleotides and the non-targeting siRNA #1 (scrambled) control. All siRNAs were resuspended at 5 μM in 1× siRNA universal buffer (Dharmacon) and stored at −20 °C. In some experiments pools of four target gene siRNA or two PARG siRNA are used and in others the pools were deconvoluted and two individual siRNA used. This is indicated in each case.

### 2.4 SiRNA transfection

For the initial siRNA screen, cells were seeded in 96-well plates (five replica wells for each individual siRNA), co-transfected with either scrambled control or individual PARG siRNA. The following day, cells were co-transfected with 20 nM siRNA (final concentration) using Dharmafect 4 reagent (Dharmacon) following manufacturer’s instructions. For further experiments, cells were co-transfected with pooled siRNA (from the four individual siRNAs) for each gene and either scrambled control or pooled PARG siRNA (from the two individual siRNAs) at a final concentration of 20 nM using Dharmafect 4 reagent (Dharmacon) following manufacturer’s instructions. Knockdown was confirmed after 48 h by real-time PCR or western blot.

### 2.5 MTT assay

Following siRNA transfection, cells were left for five days after which time, MTT (3-(4,5-Dimethylthiazol-2-yl)-2,5-DiphenyltetrazoliumBromide) (ThermoFisher) (1 mg/ml) was added to each well and the cells left for 3 h at 37 °C. The media was then aspirated off and replaced with 200 μl DMSO (Fisher scientific) and the optical density (OD) measured at 540 nm. From the five replicate wells, the highest and the lowest ODs were omitted and the average and standard deviation calculated from the remaining three replicates. This was carried out on at least two separate occasions and the average of two repeats calculated. (See supplementary material for workflow of screen analysis).

### 2.6 Clonogenic survival assay

Cells were transfected with siRNA for each of the genes of interest (as above) in 24-well plates and left for 48 h before re-plating at known densities in 90 mm dishes. When inhibitors were added to the media this was done 4 h after replating and then cells were left for 15 days to form colonies. The colonies were stained with 4% methylene blue in 70% methanol and counted.

### 2.7 RNA extraction, cDNA synthesis and real time PCR

Total RNA was extracted using the GenElute™ Mammalian Total RNA Miniprep kit (Sigma). cDNA was made using 100 μg total RNA and the Applied Biosystems High Capacity cDNA Reverse Transcriptase kit from ThermoFisher Scientific. 2 μl cDNA was mixed with SYBR Green PCR master mix (ThermoFisher Scientific) and 10 mM primers. Primers for each of the 18 genes included in the screen as well as PARG were designed to amplify between 100 bp and 150 bp cDNA transcripts.

Primers were as follows:

*BRCA1*: forward 5′-ACAGCTGTGTGGTGCTTCTGTG, Reverse − 5′-CATTGTCCTCTGTCCAGGCATC, *BRCA2*: forward 5′-GAATGCCCCATCGATTGGTCA, reverse 5′AGCCCCTAAACCCCACTTCAT, *PALB2*: forward 5′-CCTGTGCCAAAGAGAGTGAGTC, reverse 5‘AGTCTGTACCCGACCATTTCAC, *RAD51C*: forward 5′- GATGACCTGTCTCTTCGTACTC, reverse 5′-TTAGCCGTATTGTAGCAGCATG, *CHEK2*: forward 5′-GATGCTCTTGGCTGTGCAGTAC, reverse 5′-GTTCCACATAAGGTTCTCATG, *RAD51D*: forward 5′- GGCATTGGCAGTCTTGATAAAC, reverse 5′- ACATTTGCTGCCATACAGAGAC, *BRIP1*: forward 5′-ACATCGTTTGGATCATGCCCTC, reverse 5′-AACAGAGCGGATGTTCAGAATG, *BARD1*: forward 5′ −TGCCAAAGCTGTTTGATGGATG, reverse 5′-TGGTATGCGACTGTATTGATGG, *MRE11*: forward 5′-CAACCAACAAAGGAAGAGGC, reverse 5′ −TAGTGACATTTCGGGAAGGC, *NBN*: forward 5‘-TCTGTAACCAACCTGAGTCAAAC, reverse 5′-TCAAAGTTCGGGAAAAGCCATT, *RAD50*: forward 5′-TGAGGACAACAGAACTTGTGAAC, reverse 5′-TCCACGATAGGTACTTCGCC, *TP53*: forward 5′-CCCAAGCAATGGATGATTTGA, reverse 5′ −GGCATTCTGGGAGCTTCATCT, *PTEN*: forward 5′-GAAGACCATAACCCACCACAGC, reverse 5′-ATTACACCAGTTCGTCCCTTTC, *STK11*: forward 5′-CATGACTGTGGTGCCGTACTTG, reverse 5′-TTGTGACTGGCCTCCTCTTCTG, *ATM*: forward 5′-TGCTGACAATCATCACCAAGTTC, reverse 5′-TCTCCCTTCGTGTCCTGGAA, *FAM175A*: forward 5′-TCCAGCTAGTACACCACAAATC, reverse 5′-CATCTGTTTCTGGGCTGCTC, *XRCC2*: forward 5′-CCTGTGCATGGTGATATTCTTG, reverse 5′-TTCCAGGCCACCTTCTGATTTG, *CDKN2A*: forward 5′-CATAGATGCCGCGGAAGGT, reverse 5′-CCCGAGGTTTCTCAGAGCCT and *PARG*: forward 5′-GCTGTGAACCCTGCACCAAGC, reverse 5′-AAACTTTCTGATTCCGCTGTC.

U1 snRNA was used an endogenous gene control. Real-time PCR was carried out (1 × 50 °C for 2 min, 1 × 95 °C for 10 min, 40 × 95 °C for 15 s followed by 60 °C for 1 min) using the Applied Biosystems (Carlsbad, CA, USA) 7900HT fast real time PCR system. Images and data were documented using the SDS Enterprise Database software (Applied Biosystems by Life Technologies).

### 2.8 HRR assay using pDR-GFP

Cells were plated in 6-well dishes and then either transfected with siRNA as above or incubated with inhibitors for 24 h before transient transfection with 1.25 μg pDR-GFP and 1.25 μg pCMV-I*Sce*I plasmids [41] using lipofectamine 2000 (Invitrogen) following the manufacturer’s instructions. After 48 h, cells were scraped into 200 μl cold phosphate buffered saline (PBS) and analysed for GFP expression using the 488 nm laser on a FACSCalibur™ flow cytometer (BD Biosciences). Data was analysed using Flowjo software (Flowjo, LLC) and the percentage of cells expressing GFP was normalised to the non-transfected control and then calculated as the fold difference from either scrambled or non-treated cells.

### 2.9 DNA fibre analysis

Cells were seeded in 6-well plates in media containing inhibitors. After 24 h 25 μM (final concentration) of CldU (Sigma) was added to cells for 20 min at 37 °C before 250 μM IdU (final concentration, Sigma) was added for a further 20 min at 37 °C. DNA fibre analysis was then carried out as previously described [14]. Immunofluorescence was visualised using an Olympus FV1000 confocal BX61 upright microscope equipped with × 60 (1.42 NA) objective lens. Images were captured and analysed by Fluoview 3.1 software (Olympus, Shinjuku, Tokyo, Japan). Measurements of labeled tracks were performed in micrometres, by using ImageJ software (http://rsbweb.nih.gov/ij/), and converted to kilobases with the factor 1 μm = 2.59 kb.

### 2.10 Immunofluorescence

Cells were plated onto coverslips and allowed to settle before transfecting with siRNA as described above. After 48 h, medium was removed and cells washed with PBS. Cells were fixed in 4% paraformaldeyhyde solution (Insight Biotechnology) for 20 min at room temperature and then extensively washed (3 × 5 min in tris buffered saline (TBS), 1 × 10 min in PBS containing 0.5% Triton X-100 and 3 × 5 min in TBS). Coverslips were placed in 10% goat serum in TBS for 1 h at room temperature to block followed by a further 3 × 5 min washes in TBS prior to incubation with the primary antibody (anti-γH2AX ser139, Cell Signaling, 1:500 in TBS containing 3% goat serum) for 16 h at 4 °C. The coverslips were subsequently washed 4 × 10 min in TBS followed by incubation with the secondary antibody, (Alexa fluor 594 goat anti-rabbit IgG, Fisher, 1:500 in TBS containing 3% goat serum) for 1 h at room temperature and finally washed 3 × 5 min TBS. Coverslips were then mounted onto microscope slides with DAPI containing mountant (Vector Labs). Images were obtained with a Zeiss LSM 510 inverted confocal microscope using planapochromat 63 × /NA 1.4 oil immersion objective and excitation wavelengths 488 nm, 546 nm and 630 nm. Through focus maximum projection images were acquired from optical sections 0.5 μM apart and with a section thickness of 1.0 μm. Images were processed using Adobe Photoshop (Abacus Inc.). The frequency of cells containing γH2AX foci was determined by counting at least 100 nuclei on each slide.

### 2.11 Western blotting

Cells were lysed in RIPA buffer (20 mM Tris-HCl (pH 7.5) 150 mM NaCl, 1 mM Na_2_EDTA, 1 mM EGTA, 1% NP-40, 1% sodium deoxycholate, 2.5 mM sodium pyrophosphate, 1 mM, b-glycerophosphate, 1 mM Na_3_VO_4_,1 μg/ml leupeptin) in the presence of 1× protease and phosphatase inhibitor cocktails (Roche). An aliquot of 30 μg total protein was run on an SDS-PAGE gel and transferred to Hybond ECL membrane (GE Healthcare). This membrane was immunoblotted with antibodies against Poly(ADP-ribose) (1:400, 10H Enzo Life Sciences), PARG (1:250, Abcam), PARP1 (1:1000, Santa Cruz), BRCA1 (1:500, Santa Cruz), BARD1 (1:500, H-300, Santa Cruz), PTEN (1:1000, Cell Signaling), PALB2 (1:500, Novus Biologicals), FAM175A (1:1000, Bethyl) and β-tubulin (1:2000, Sigma) each diluted in 5% milk and incubated at 4 °C overnight. After the addition of the appropriate HRP-conjugated secondary antibody and further washes, the immunoreactive protein was visualised using ECL reagents (GE Healthcare) following manufacturer’s instructions.

## 3 Results

### 3.1 An siRNA screen for synthetic lethality with PARG depletion

**Fig. 1:**
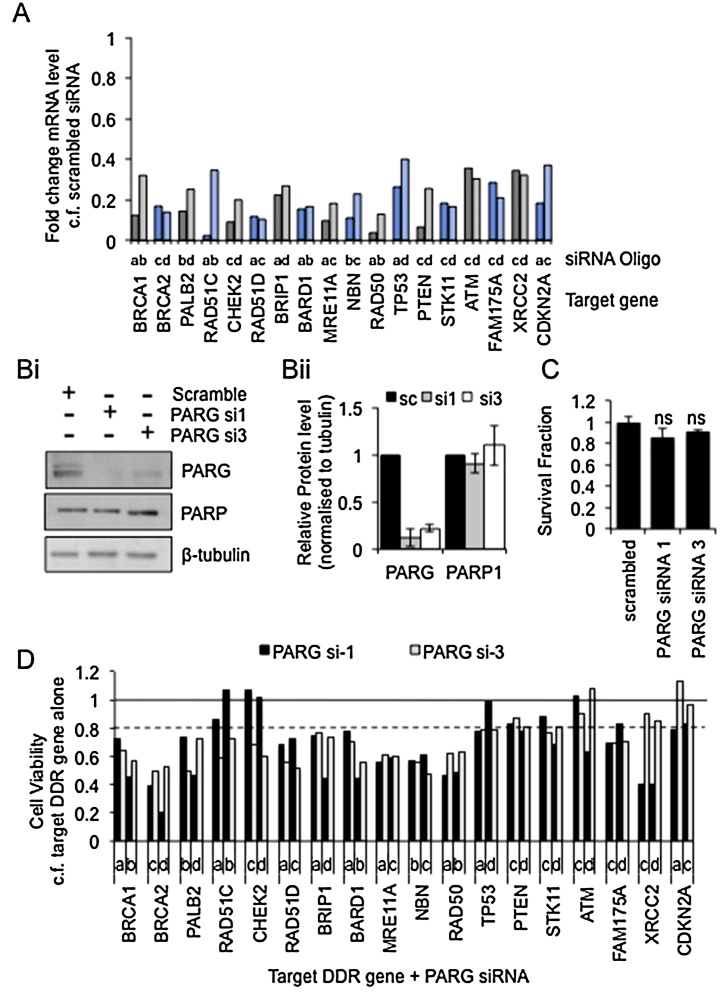
Synthetic lethal screen using siRNA. (A) Relative expression of mRNA for each test gene compared to scrambled control 24 h post transfection as measured by qRT-PCR and normalised to U1 snRNA. Letters above target gene names refer to separate oligos (a–d) for each gene. (B) Protein expression of PARG and PARP 48 h post transfection with each PARG siRNA, representative western blot and mean and standard deviation of quantification of three independent repeats are shown. (C) MCF7 cell viability 5 days post transfection with each PARG siRNA measured by MTT assay, mean and standard deviation of four independent repeats are shown. (D) MCF7 cell viability as measured by MTT assay 5 days post transfection with combinations of individual siRNA oligos. For each target DDR gene siRNA + PARG siRNA survival fraction is calculated compared to DDR gene alone, mean of two independent repeats is shown.

Previously PARG depletion or inhibition was shown to be synthetic lethal with depletion of BRCA2 [39]. Here, this work is extended to examine the synthetic lethal interactions of PARG with a range of known or putative DNA damage response (DDR) genes. Each of these genes has also been associated with breast cancer and the screen was performed in the breast cancer cell line MCF7 [42]. Four siRNAs against each target DDR gene were tested (oligos a–d – data not shown) and the two that reduced expression of their target mRNA the most (by at least 60%) were taken forward (Fig. 1A). Two separate siRNAs against PARG were also used and shown to reduce expression of PARG protein by 80–90% without significant change in PARP1 protein levels (Fig. 1B). Transfection with PARG siRNA alone did not significantly alter the survival of MCF7 cells (Fig. 1C). To initially examine synthetic lethality, combinations of each target DDR gene siRNA with each PARG siRNA were transfected into MCF7 cells along with the relevant controls and cell viability determined using an MTT assay (workflow in supplementary material). Depletion of each of the target DDR genes alone had a varied effect on cell viability (supplementary Fig. 1), therefore after normalizing for the effect of PARG depletion, the viable fraction of cells depleted of target DDR gene plus PARG was calculated relative to the viability of cells depleted of the corresponding target DDR gene alone. Although a reduction in viability is not definitive of synthetic lethality (cell death), it does indicate genetic interactions and therefore in this initial screen a potential synthetic lethal interaction was considered to exist if three or more of the four combinations of target DDR gene/PARG siRNA showed at least 20% reduction in viability compared to controls (Fig. 1D). By these criteria disruption of BRCA1, BRCA2, PALB2, RAD51D, BRIP1, BARD1, MRE11, NBN, RAD50, TP53, and FAM175A can be considered synthetic lethal with PARG depletion.

### 3.2 **A** chemical screen for genetic sensitivities to PARG inhibition

**Fig. 2:**
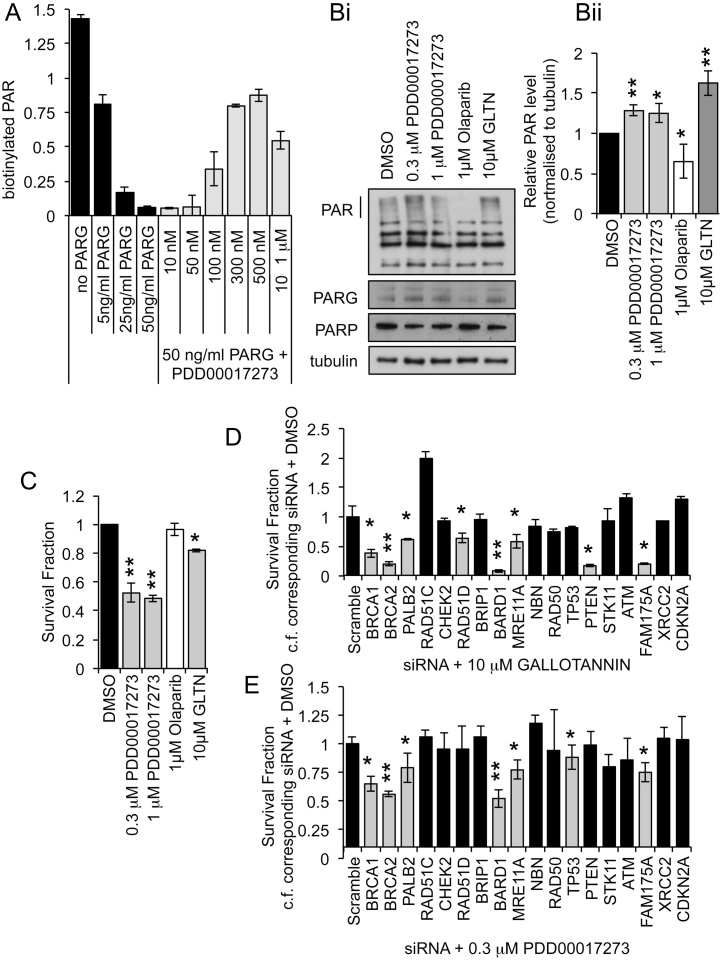
Synthetic lethal screen using PARG inhibitors. (A) Quantification of PARG activity in the presence of increasing concentrations of PDD00017273 as measured by the ability of recombinant PARG to hydrolyse biotinylated PAR polymers from histones. (Bi) Western Blot for poly(ADP-ribose) (PAR), PARP1, PARG and tubulin, in MCF7 cells control (DMSO), treated with PARG inhibitors (Gallotannin (GLTN) and PDD00017273) or PARP inhibitor (Olaparib). (Bii) Quantification represents mean intensity of PAR over three independent repeats relative to tubulin loading control. (C) Survival fraction of MCF7 cells untreated (DMSO), treated with PARG inhibitors (GLTN and PDD00017273) or PARP inhibitor (Olaparib) as measured by clonogenic survival assay, mean and standard deviation of three independent repeats are shown. Statistical significance was calculated using the Student’s *T*-test, compared to DMSO control. (D) Survival fraction of pooled target DDR gene siRNA transfected MCF7 cells treated with PARG inhibitors Gallotannin (GLTN) (E) PDD00017273 compared to corresponding siRNA transfected DMSO treated cells. Survival was measured by clonogenic survival assay; mean and standard deviation of three independent repeats are shown. Statistical significance was calculated using the Student’s *T*-test, comparing DDR gene + inhibitor to scrambled control + inhibitor where * = p < 0.05, ** = p < 0.01.

Previously, Gallotannin was used to inhibit PARG showing complete inhibition of PAR polymer degradation at 10 μM [39]. However there are concerns over the specificity of Gallotannin [43], [44]. We therefore also used a newly identified specific PARG inhibitor PDD00017273 [40]. To confirm PARG inhibition by this new agent, recombinant PARP1 was used to add biotinylated PAR to histones; recombinant PARG was then added and loss of PAR in the presence or absence of PDD00017273 was assessed (Fig. 2A). PDD00017273 caused a dose dependent inhibition of PARG with significant inhibition being seen between 0.1 and 1 μM. This is consistent with previous reports of its activity [40], [45]. To determine whether PDD00017273 also inhibits PARG in cells, the MCF7 cell line was treated for 24 h with 0.3 μM and 1 μM PDD00017273 in the absence of any exogenous DNA damage. Western blotting of the resultant cell lysates revealed an increase in PAR polymers at 0.3 μM (Fig. 2B), confirming that PDD00017273 does indeed inhibit degradation of PAR polymers in cells. Surprisingly, in the recombinant protein assay less PARG inhibition was seen at 1 μM than 0.3 μM PDD00017273. In western blotting, quantification of three repeats did not demonstrate this difference, but close examination of each repeat revealed that PARG inhibition was less consistent at the higher dose (Supplementary data S2A). Cells treated with 10 μM Gallotannin or the PARP inhibitor Olaparib for 24 h showed the expected increase and reduction of PAR polymers respectively (Fig. 2B). Consistent with our previous findings, PARG inhibition alone reduced MCF7 cell survival in long-term clonogenic survival assays, while PARP inhibition had no significant effect on survival (Fig. 2C). The same panel of target DDR gene siRNAs were then used to determine the effect of gene deletion on PARG inhibitor sensitivity. The individual siRNAs for each gene were pooled then each experiment was repeated in triplicate on three separate occasions. Survival was determined by clonogenic survival assay in MCF7 cells using 10 μM Gallotannin or 0.3 μM PDD00017273, as these doses gave maximum inhibition with the lowest toxicity (Fig. 2A–C) [39]. To take account of the effect of depleting each gene, survival fractions were calculated as the fraction of cells surviving following treatment with DDR gene siRNA and inhibitor compared to DDR gene siRNA alone (Fig. 2D and E). Compared to similarly treated scrambled siRNA treated control cells, decreased survival was seen in BRCA1, BRCA2, PALB2, FAM175A, and BARD1 depleted cells following treatment with 10 μM Gallotannin or 0.3 μM PDD00017273. Depletion of MRE11A increased sensitivity only to PDD00017273, while PTEN and RAD51D depletion sensitized only to Gallotannin. Under the same experimental conditions, survival was reduced in BRCA1, BRCA2, PALB2, RAD51C, BARD1, MRE11A, RAD50, ATM, FAM175A and XRCC2 depleted MCF7 cells treated with the PARP inhibitor Olaparib (supplementary data S2B).

### 3.3 An siRNA screen for increased DNA damage with PARG depletion

**Fig. 3:**
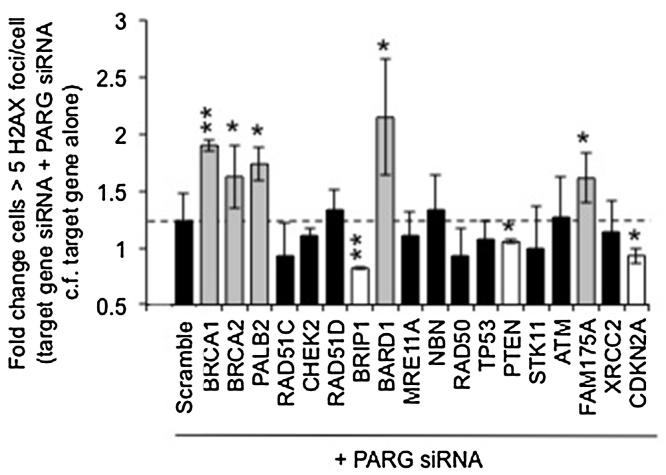
γH2AX formation screen with siRNA. Fold increase in cells with greater than five γH2AX foci/cell in MCF7 cells 24 h post transfection with combinations of pools of target DDR gene siRNA plus pooled PARG siRNA compared to corresponding target DDR gene alone. 100 cells were counted on three separate occasions; mean and standard deviation are shown. Statistical significance was calculated using the Student’s *T*-test, compared to scrambled siRNA + PARG siRNA control, where * = p < 0.05, ** = p < 0.01. Representative images are shown in Supplementary data S3.

The synthetic lethal effects of PARG with BRCA2 are reported as due to increased levels of unrepaired DNA damage in cells. The formation of γH2AX foci is a general marker of DNA damage. SiRNA mediated depletion of BRCA1, BRCA2, PALB2, FAM175A, and BARD1 combined with PARG siRNA resulted in increased γH2AX foci compared to depletion of each DDR gene alone (Figs. 3 and S3 ), suggesting increased levels of DNA damage occur and/or that repair is reduced when PARG expression is reduced in these genetic backgrounds. Interestingly, depletion of PARG in a BRIP1, PTEN or CDKN2A depleted background resulted in significantly fewer γH2AX foci than when BRIP1, PTEN or CDKN2A where depleted alone, suggesting a different functional relationship between these genes.

### 3.4 BRCA1, BRCA2, PALB2, FAM175A and BARD1 depleted cells are confirmed as sensitive to PARG depletion and inhibition

**Fig. 4:**
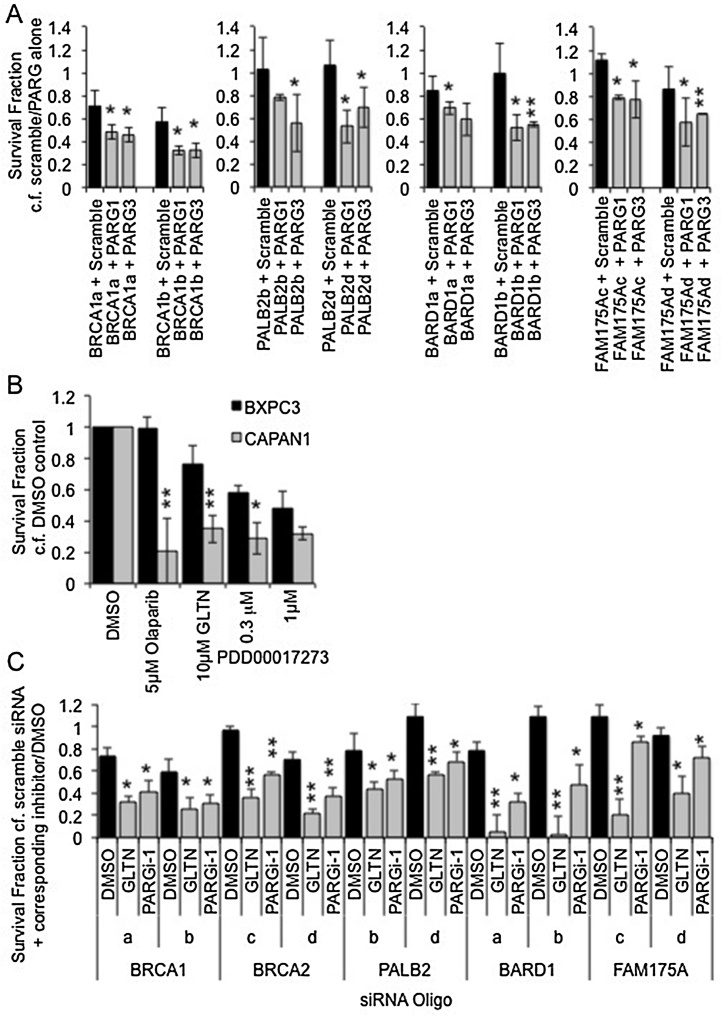
Confirmation of synthetic lethal relationships by clonogenic survival assay. (A) Survival fraction of MCF7 cells as measured by clonogenic survival assay 14 days post transfection with siRNA. Combinations of two different siRNA targeting PARG (PARG1 or PARG3) are combined with two different siRNA targeting the test DDR gene (indicated by a letter after the test gene name). For each DDR gene siRNA + scrambled/PARG siRNA survival fraction is calculated compared to corresponding scrambled/PARG siRNA alone. Statistical significance was calculated using the Student’s *T*-test, comparing DDR gene + PARG gene siRNAs to DDR gene + scrambled control siRNAs. (B) Survival fraction of wildtype (BXPC3) and BRCA2 deficient (CAPAN1) cells treated with DMSO, PARP inhibitor (olaparib), or the PARG inhibitors (gallotannin (GLTN) and PDD00017273), as measured by clonogenic survival assay. Statistical significance was calculated using the Student’s *T*-test, comparing BXPC3 to CAPAN1 for each treatment. (C) Survival fraction of MCF7 cells as measured by clonogenic survival assay 14 days post transfection with DDR gene siRNA with addition of the PARG inhibitors Gallotannin (GLTN) or PDD00017273. For each DDR gene siRNA + DMSO/inhibitor survival fraction is calculated compared to corresponding DMSO/inhibitor + scrambled siRNA. Statistical significance was calculated using the Student’s *T*-test, comparing PARG inhibitor treated cells to respective DMSO control. In each case mean and standard deviation of three independent repeats are shown. * = p < 0.05, ** = p < 0.01.

There were just five DDR genes that resulted in decreased cell viability in response to PARG siRNA, and decreased survival with Gallotannin and PDD00017273 – *BRCA1, BRCA2, PALB2, FAM175A* and *BARD1* (Table 1). Significantly, these were the only genes in the screen that resulted in upregulated γH2AX foci formation (Table 1). BRCA1, BRCA2, PALB2, FAM175A and BARD1, were therefore examined further. During the PARG siRNA screening process, cell viability was analysed by MTT assay. To validate these findings, combinations of two different individual target DDR gene siRNAs with each of two different individual PARG siRNAs were transfected into MCF7 cells along with the relevant controls, and cell survival determined by clonogenic survival assay. The experiment was repeated on three separate occasions and statistical significance calculated. The efficiency of siRNA mediated depletion was ensured by western blotting (Supplementary data S4). For each gene there was a reduction in protein expression that was accompanied by a reduction in survival in at least three of four combinations of PARG siRNA with target DDR gene siRNA compared to target DDR gene alone (Fig. 4A). For BRCA2 a synthetic lethal relationship has previously been demonstrated using siRNA, therefore here the CAPAN-1 cell line, which carries a naturally occurring 6174delT mutation in one *BRCA2* allele accompanied by loss of the wild-type allele was used to demonstrate further the clinical potential of PARG inhibitors (Fig. 4B). Compared to the control BRCA2 wildtype BXPC3 cells [46], CAPAN-1 cells were more sensitive to both Gallotannin and PDD00017273.

During the inhibitor screening process (Fig. 2) target DDR gene siRNA were pools of individual siRNAs; therefore to further validate these results each of two different target DDR gene siRNAs were used in combination with Gallotannin and PDD00017273 and survival assayed by clonogenic assay on three separate occasions (Fig. 4C). Depletion of each of the five DDR genes resulted in significant reduction in cell survival compared to controls.

These data together confirm the validity of PARG as a mono-therapeutic target in BRCA1, BRCA2, PALB2, FAM175A and BARD1 depleted cells.

### 3.5 PARG inhibition/depletion stalls replication forks and induces DNA damage that requires HR for repair

**Fig. 5:**
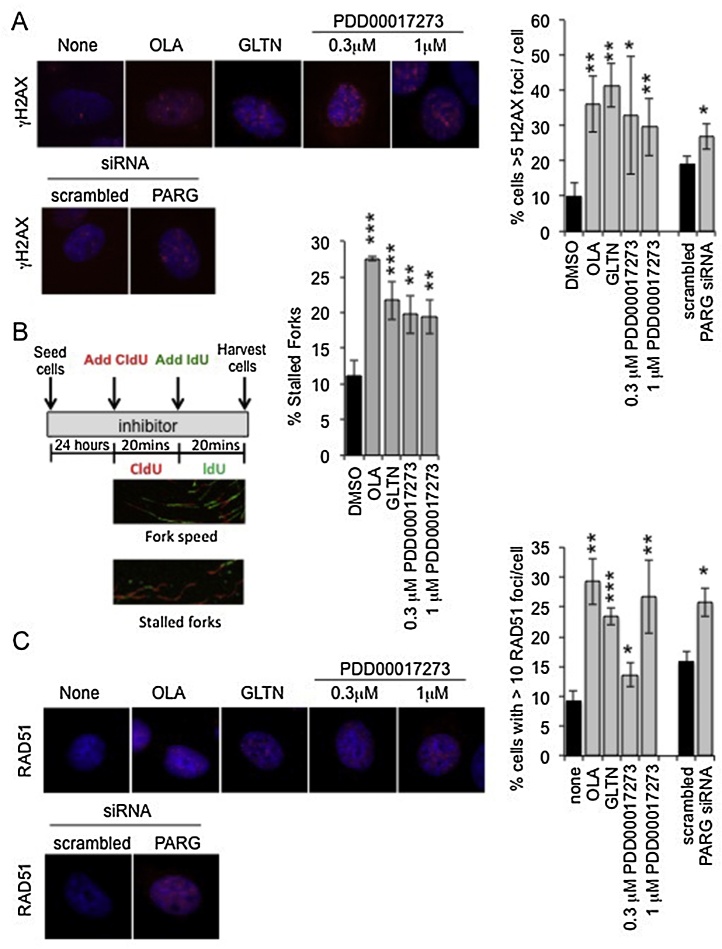
PARG depletion/inhibition disrupts replication forks and induces accumulation of γH2AX and RAD51 foci. (A) Percentage of cells with greater than five γH2AX foci/cell in MCF7 cells 24 h post treatment with control (DMSO), PARP inhibitor (Olaparib (OLA)), the PARG inhibitors (gallotannin (GLTN) and PDD00017273), scrambled siRNA and PARG siRNA (a pool of PARG1 + PARG3 siRNA). Example images are shown. Statistical significance was calculated using the Student’s *T*-test, compared to DMSO or scrambled control, where * = p < 0.05, ** = p < 0.01, *** = p < 0.001. (B) DNA fibre analysis of replication fork stalling in MCF7 cells treated with PARP and PARG inhibitors. Cells were incubated in inhibitor and then pulse labeled with CldU for 20 min, and then labeled switched to IdU for 20 min. Fork stalling was calculated as a percentage of CIdU only labeled tracts (red) from continuous forks (CIdU (red) and IdU (green) labeled tracts). Example images of replication forks are shown. At least 100 forks were counted on each of three separate occasions. Data bars present the mean and standard deviation of three independent experiments. Statistical significance was calculated using the Student’s *T*-test compared to DMSO control. (C) Percentage of cells with greater than 10 RAD51 foci/cell in MCF7 cells 24 h post treatment with control (DMSO), PARP inhibitor (Olaparib (OLA)), the PARG inhibitors (Gallotannin (GLTN) and PDD00017273), scrambled siRNA and PARG siRNA (a pool of PARG1 + PARG3 siRNA). Example images are shown. Statistical significance was calculated using the Student’s *T*-test, compared to DMSO or scrambled control.

Gallotannin has previously been shown to increase γH2AX foci formation. These foci were reduced in cells co-treated with the replication inhibitor aphidicolin [39], and thus the foci were considered to be due to DNA aberrations at replication forks. Synthetic lethality was therefore suggested to be the result of a lack of BRCA2 for repair/restart of the replication forks. Consistent with this, Gallotannin, PDD00017273 and PARG siRNA all induced γH2AX foci formation compared to the respective controls (Fig. 5A). Previously, we have shown that inhibition of PARP caused replication fork stalling [14], thus the progression of single replication forks was examined using a DNA fibre assay. In this assay, inhibition of PARG resulted in increased replication fork stalling (Fig. 5B) directly demonstrating an effect of PARG inhibition on replication forks. Consistent with an increased requirement for certain HRR proteins following inhibition or depletion of PARG, Gallotannin, PDD00017273 and PARG siRNA all increased formation of RAD51 foci compared to the respective controls (Fig. 5C).

### 3.6 BRCA1, BRCA2, PALB2, FAM175A and BARD1 are all required for HRR following PARG depletion

**Fig. 6:**
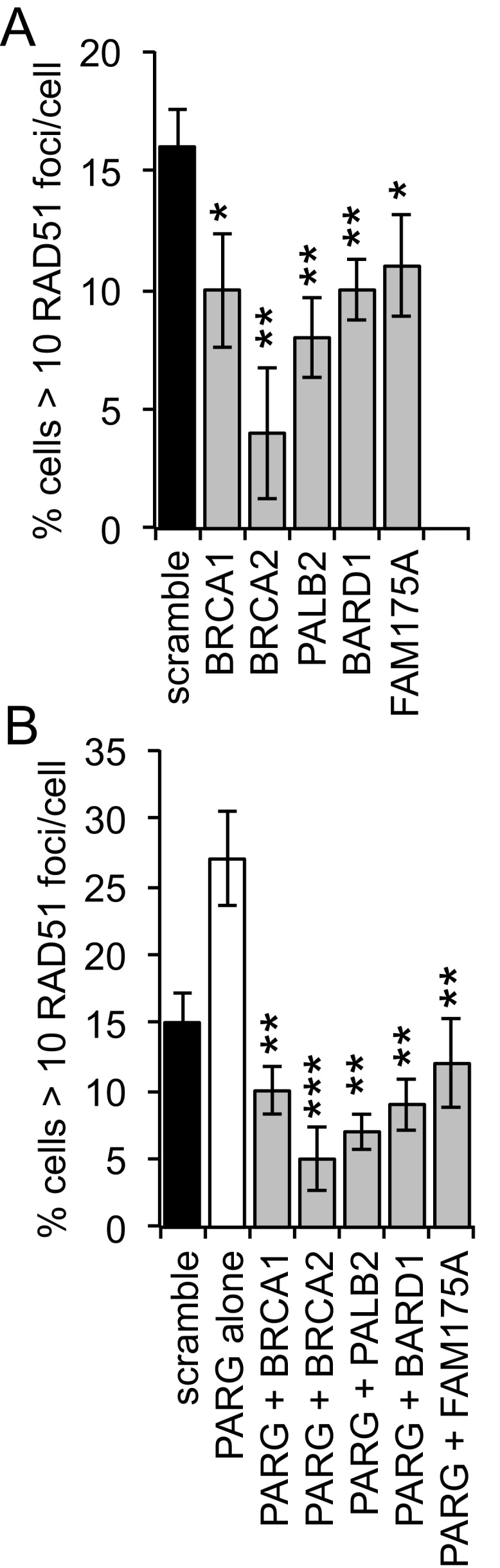
Homologous recombination after PARG depletion. (A) Percentage of cells with greater than 10 RAD51 foci/cell in MCF7 cells 24 h post treatment with scrambled or target DDR gene siRNA. (B) Percentage of cells with greater than 10 RAD51 foci/cell in MCF7 cells 24 h post treatment with scrambled, PARG siRNA (a pool of PARG1 + PARG3 siRNA) and PARG + target DDR gene siRNA. For each 100 cells were counted on three separate occasions and mean and standard deviation shown. Statistical significance was calculated using the Student’s *T*-test, compared to PARG siRNA alone control, where * = p < 0.05, ** = p < 0.01, *** = p < 0.001.

The function of BRCA1, BRCA2, PALB2, FAM175A, and BARD1 in HRR was demonstrated by a reduction in the number of endogenous RAD51 foci seen in cells (Fig. 6A) and confirmed using a GFP reporter assay (supplementary Fig. S5). Further, PARG depletion-induced RAD51 foci formation was reversed when cells were co-depleted of PARG and BRCA1, BRCA2, PALB2, FAM175A, or BARD1 (Fig. 6B), again suggesting that cell death is due to a lack of repair of DNA damage/inability to restart stalled replication forks. Interestingly, while PARG depletion and inhibition induced Rad51 foci formation in the absence of exogenous DNA damage (Figs. 5 E and 6 B), it did not increase HRR at an I-*Sce*I induced double strand break (Fig. 6A), indicative of the difference in response of cells to various types of DNA lesions.

Together these data support our previous findings and a model whereby inhibition or depletion of PARG leads to fork stalling and fork aberrations, resulting in signalling and recruitment of HRR proteins for repair. Therefore in the absence of these HRR proteins, PARG depleted or inhibited cells cannot survive.

## 4 Discussion

Our use of a novel class of PARG inhibitor to selectively kill tumour cells with particular genetic defects, combined with our data generated using Gallotannin and PARG siRNA support the future development of PARG inhibitors as mono-therapeutic agents. This is the first such report of a screen for synthetic lethal interactions (or more correctly, induced cell death) with PARG and the first potential synthetic lethal use for the novel PARG inhibitor PDD00017273 [40]. It is worth noting that the PDD00017273 induced reduction in long-term survival seen here in MCF7 cells, is in contrast to that seen with the same agent in a short-term assay in HeLa cells [40], this could be due to cellular specificity or the long-term versus short-term effects of the drug.

Here, BRCA1, BRCA2, PALB2, FAM175A, and BARD1 were all seen to be synthetic lethal with PARG following siRNA mediated depletion and inhibition of PARG activity, each gene has been associated with breast cancer and ovarian cancer [47], [48], [49], [50], as well as prostate cancer (*BRCA1, BRCA2, PALB2*) [50], [51], [52], [53] and pancreatic cancer (*PALB2, BRCA1, BRCA2*) [54], [55], [56], [57] thus PARG inhibition may have clinical potential in some of these patients. However, another report using PARG siRNA in a different cellular background fails to confirm that BRCA1 defects lead to sensitivity to PARG depletion [58], perhaps highlighting the importance of other genetic factors. Indeed MCF7 cells have over 30 known nonsynonymous mutations including *DNA-PK (PRKDC)* and *ERCC6*. It would therefore be interesting to examine if these synthetic lethal relationships hold true for other cancer cell lines or other disease areas. In addition, given the breast cancer focused screen performed here, a full screen of synthetic lethality with PARG inhibitors is warranted. The full clinical potential of PARG inhibitors will be the subject of future investigations.

While BRCA1, BRCA2 and PALB2 have been associated with HRR at DNA double strand breaks and replication fork- associated DNA damage [59], [60], [61], the role of FAM175A and BARD1 are less well described. FAM175A (ABRAXAS) has been implicated in the repair of ionizing radiation and crosslink induced DNA damage, but it is reported as not being involved in response to the replication inhibitor hydroxyurea [62]. Our FAM175A and BARD1 data demonstrate reduced I-*Sce*I induced HRR and reduced RAD51 foci formation following PARG depletion, supporting a function for each at both double strand breaks and replication associated DNA lesions.

In previous studies the relative degree of sensitivity to PARP or PARG inhibition was different between cell lines [40], perhaps indicative of a different pharmacology in different genetic backgrounds. Here in parallel to PARG inhibitor screening, sensitivity to the PARP inhibitor Olaparib was carried out, allowing direct comparison of the role of various DDR genes in sensitivity to PARG inhibition within the same genetic background. When comparing PARP and PARG synthetic lethal interactions, we saw that many of the same genes are implicated (e.g. *BRCA1, BRCA2, PALB2, BARD1*, and *FAM175A*), likely reflecting the function of PARG to reverse PARP activity and allow repair to proceed. In contrast, RAD51C, ATM, and XRCC2 depleted cells were sensitive only to PARP inhibition, and showed no effect with PARG siRNA, Gallotannin or with PDD00017273. The precise reasons for these differences are not clear but they do argue that not all functions of PARP and PARG are common. Perhaps it is worth noting that unlike BRCA1/2, RAD51C and XRCC2 are thought to act late in HRR, downstream of RAD51 foci formation. Using siRNA, PTEN has previously been reported as not having a synthetic lethal relationship with PARG [58], here we confirm this with a PARG inhibitor. Interestingly though, co-depletion of BRIP1 or PTEN and PARG resulted in significantly fewer γH2AX foci compared to BRIP1/PTEN alone, suggesting that BRIP1 and PTEN may have a complex functional interaction with PARG, which maybe important for future understanding of the function of PARG during DNA damage repair.

Importantly, PARG inhibition/depletion was capable of killing BRCA1, BRCA2, PALB2, FAM175A, or BARD1 depleted cells in the absence of any exogenous DNA damaging agents. We previously demonstrated that the increase in DNA damage signalling following PARG inhibition was dependent on active replication [39]. Furthermore, PARG is essential for recovery from long-term replication stress [38]. Thus, it is likely that the function of HRR proteins is to restore replication following PARG inhibition, however the nature of PARG-induced replication stress and the role of HRR proteins in this process is less clear. Additionally, we have previously argued that the reason for synthetic lethality is an inability to undergo HRR for collapsed replication fork restart [39]. HRR is known to be required to restart collapsed replication forks [63], and the poly(ADP-ribosyl)ation activity of PARP1 is known to stabilize DNA replication forks [64], [65]. PARG depletion/inhibition has been seen to increase the number of endogenous reversed replication forks and post-replicative single strand DNA breaks [37], raising the possibility that in the absence of PARG, HRR could also have a function in restarting these reversed forks. Consistent with either hypothesis is our finding that PARG inhibition in the absence of any exogenous damage stalls the replication fork and that endogenous RAD51 foci are induced following PARG inhibition or depletion. The nature of the DNA lesion/s induced by PARG inhibition/depletion and the function of various DNA repair proteins (including PARP and PARG themselves) has been and is likely to continue to be a subject of debate.

While depletion/inhibition of either PARG or PARP increases endogenous RAD51 foci formation, PARG depletion/inhibition results in decreased HRR at a site-specific DNA double strand break, which is in contrast to the finding that HRR at the same site in PARP-inhibited cells is normal [66]. The reason for the difference between PARP and PARG depletion is possibly because PARP plays a regulating role rather than essential role in HRR, thus its absence does not prevent HRR. However, irreversible binding of PAR to DNA double strand breaks is likely to result in failure of the ability of other proteins to complete HRR.

## 5 Conclusion

In summary, we show that disruption of the HRR associated proteins BRCA1, BRCA2, PALB2, FAM175A and BARD1 are synthetic lethal with depletion or inhibition of PARG. PARG inhibition stalls replication forks, triggers DNA damage signalling, and HRR protein accumulation in repair foci, suggestive of a function of these proteins in replication fork restoration. Although further testing is needed to validate findings in other cellular backgrounds and in pre-clinical studies, our data do suggest that the future development of clinically applicable PARG inhibitors may hold promise for treatment of some types of HRR-deficient tumours.

## 6 Funding

This work was supported by Breast Cancer Now [grant number PR016], and CRUK grant [number C5759/A17098].

## 7 Author contributions

HB conceived, designed and initiated the study. PG and EG performed the experiments. DJ and KS designed and synthesised PDD00017273. PG and HB wrote the manuscript. All authors discussed the results and commented on the manuscript.

## Figures and Tables

**Table 1:** Summary of results following depletion of target DDR gene using siRNA in combination with PARG siRNA or PARG inhibitors.

TEST DDR GENE	Reduced cell viability + PARG siRNA	Reduced cell survival + GALLOTANNIN	Reduced cell survival + PDD00017273	Increased γH2AX
BRCA1	Yes	Yes	Yes	Yes
BRCA2	Yes	Yes	Yes	Yes
PALB2	Yes	Yes	Yes	Yes
RAD51C	No	No	No	No
CHEK2	No	No	No	No
RAD51D	Yes	Yes	No	No
BRIP1	Yes	No	No	Reduced
BARD1	Yes	Yes	Yes	Yes
MRE11A	Yes	No	Yes	No
NBN	Yes	No	No	No
RAD50	Yes	No	No	No
TP53	Yes	No	No	No
PTEN	No	Yes	No	Reduced
STK11	No	No	No	No
ATM	No	No	No	No
FAM175A	Yes	Yes	Yes	Yes
XRCC2	No	No	No	No
CDKN2A	No	No	No	Reduced
