# Supplementary material for: Specific killing of DNA damage-response deficient cells with inhibitors of poly(ADP-ribose) glycohydrolase
Source: DNA Repair (Amst). 2017 Apr;52:81–91. doi: 10.1016/j.dnarep.2017.02.010 (PMC5360195; doi:10.1016/j.dnarep.2017.02.010)
Supplement: Supplementary file 1 [file mmc1.docx]

**Synthetic Lethal cell viability screen workflow**

1. Reverse transfection of MCF7 cells with one of 2 siRNA (PARG1 and PARG3) against PARG in combination with each of 2 siRNA against test DDR genes (a-d) and relevant controls was set up as below (5 replicates for each combination)

Where only one siRNA was used the final concentration was made up to 20nM using scrambled siRNA.

2. Leave for 5 days

3. Measure cell survival by MTT assay.

4. Average of 5 technical repeats in each experiment calculated

5. Data normalized to scrambled siRNA control (well A) (each siRNA treatment/scrambled siRNA alone).

6. Data normalized for effect of depletion of the respective PARG gene alone

(target gene siRNA + PARG siRNA)/PARG siRNA alone – i.e. wells E and H / B and wells F and I / C.

7. This was used to compare survival after depletion with corresponding target gene siRNA alone

Survival fraction = normalized target gene siRNA+PARG siRNA/target gene siRNA alone. (E and F compared to D and H and I compared to G).

A synthetic lethal relationship was considered to be present in the screen if once the effect of PARG siRNA alone had been eliminated at least ¾ of the combinations of PARG+test gene siRNA showed greater that 20% reduction in survival compared to the corresponding test gene siRNA alone.

**Supplementary figure legends**

S1

MCF7 cell viability five days post transfection with each target DDR gene siRNA compared to scrambled siRNA measured by MTT assay, mean and SEM of two independent repeats are shown.

S2

A: Three repeats of western blotting for PAR following incubation with PARG or PARP inhibitors. Each repeat is quantified relative to tubulin.

B: Survival fraction of target DDR gene siRNA transfected MCF7 cells treated with the PARP inhibitor Olaparib (1 μM). Survival was measured by clonogenic survival assay, mean and standard deviation of three independent repeats are shown. Statistical significance was calculated using the Student’s T-test, compared to drug + scrambled control.

S3

γH2AX foci following treatment with pooled target DDR gene siRNA with or without pooled PARG gene siRNA

S4

Western blot to demonstrate depletion of target DDR genes and PARG following 48 h treatment with corresponding siRNA

S5

Relative recombination frequency. The DR-GFP reporter consists of two defective *GFP* genes. Expression of I-*Sce*I endonuclease results in a double-strand break (DSB) at the I-*Sce*I site in the *SceGFP* gene which can be repaired using the homologous sequence in the *iGFP* gene to generate GFP positive cells which are quantified by flow cytometry. The mean and standard deviation of percentage GFP positive cells in MCF7 cells 24 hours post transfection with siRNA as indicated are shown. Statistical significance was calculated using the Student’s T-test, compared to scrambled control, using three independent repeats.
