# Supplementary figures and images for: Specific killing of DNA damage-response deficient cells with inhibitors of poly(ADP-ribose) glycohydrolase
Source: DNA Repair (Amst). 2017 Apr;52:81–91. doi: 10.1016/j.dnarep.2017.02.010 (PMC5360195; doi:10.1016/j.dnarep.2017.02.010)

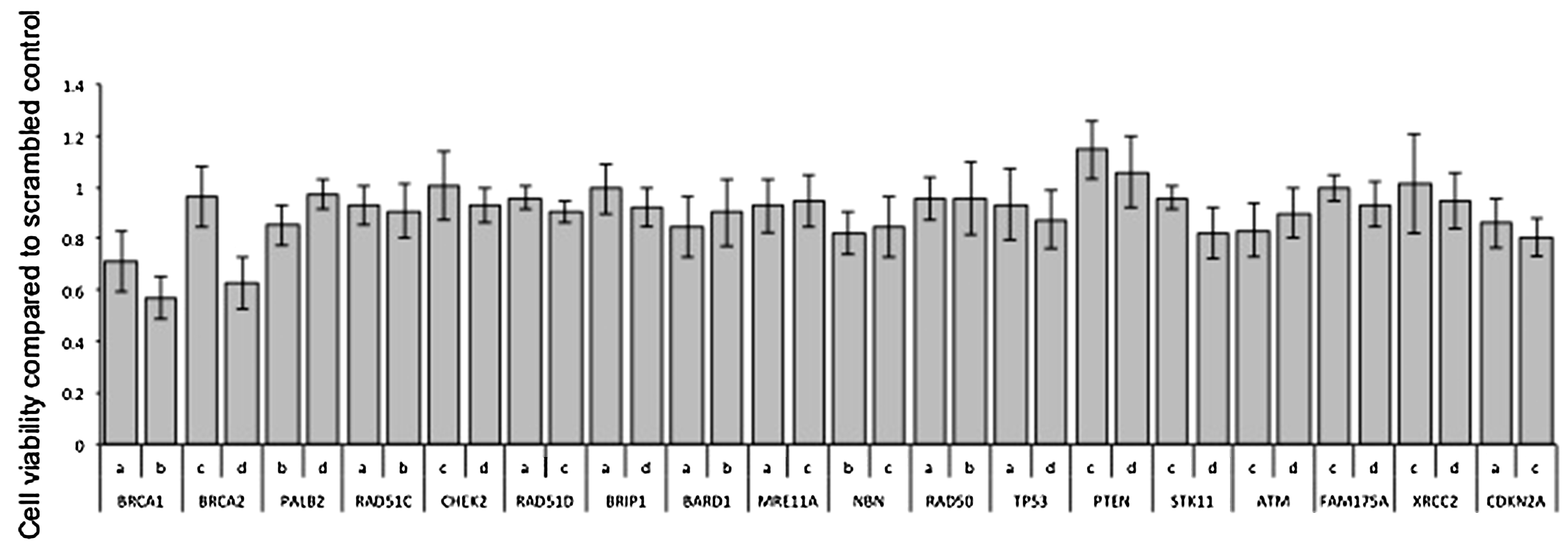

Supplement: Supplementary file 2 [file mmc2.jpg]

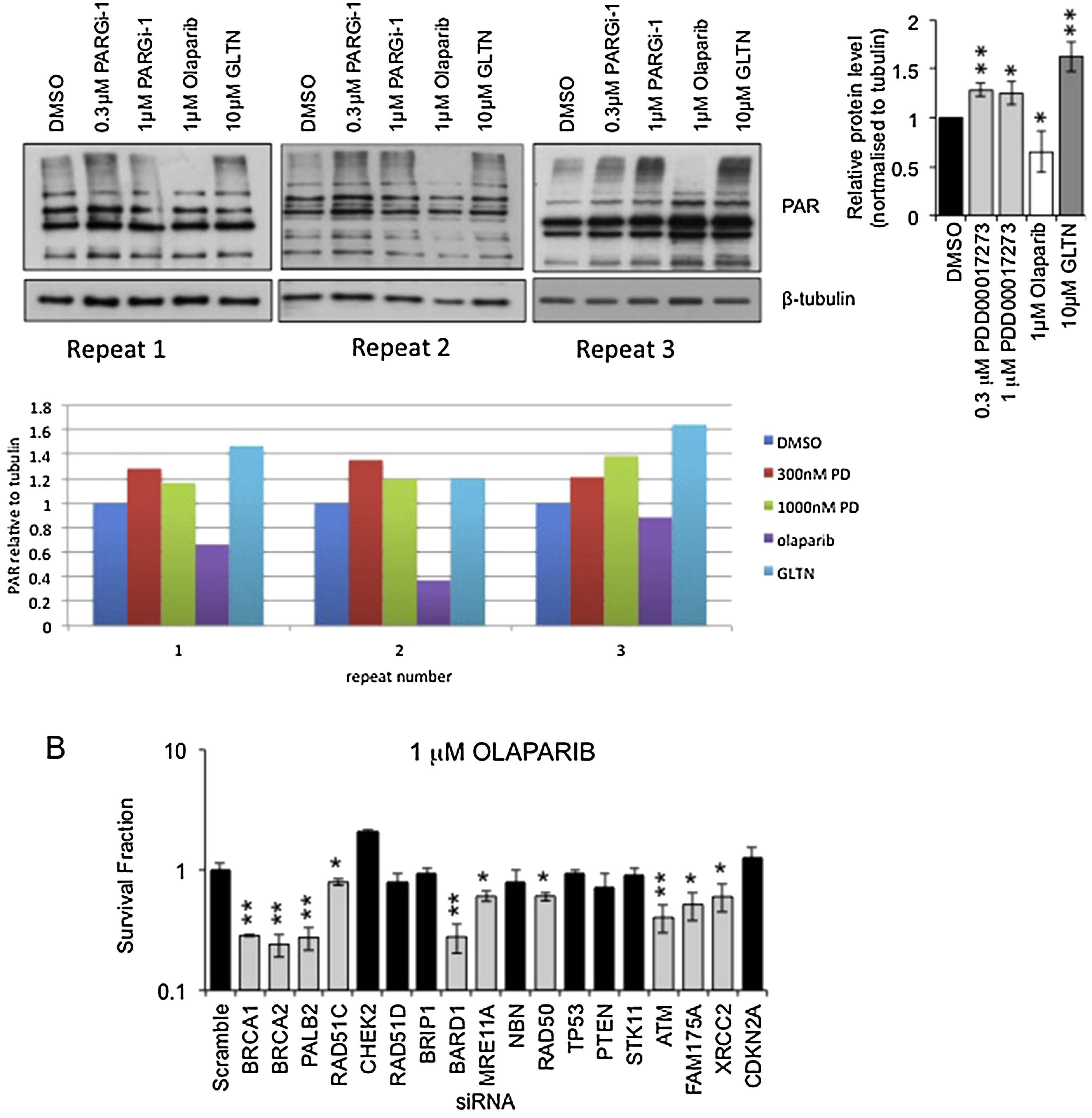

Supplement: Supplementary file 3 [file mmc3.jpg]

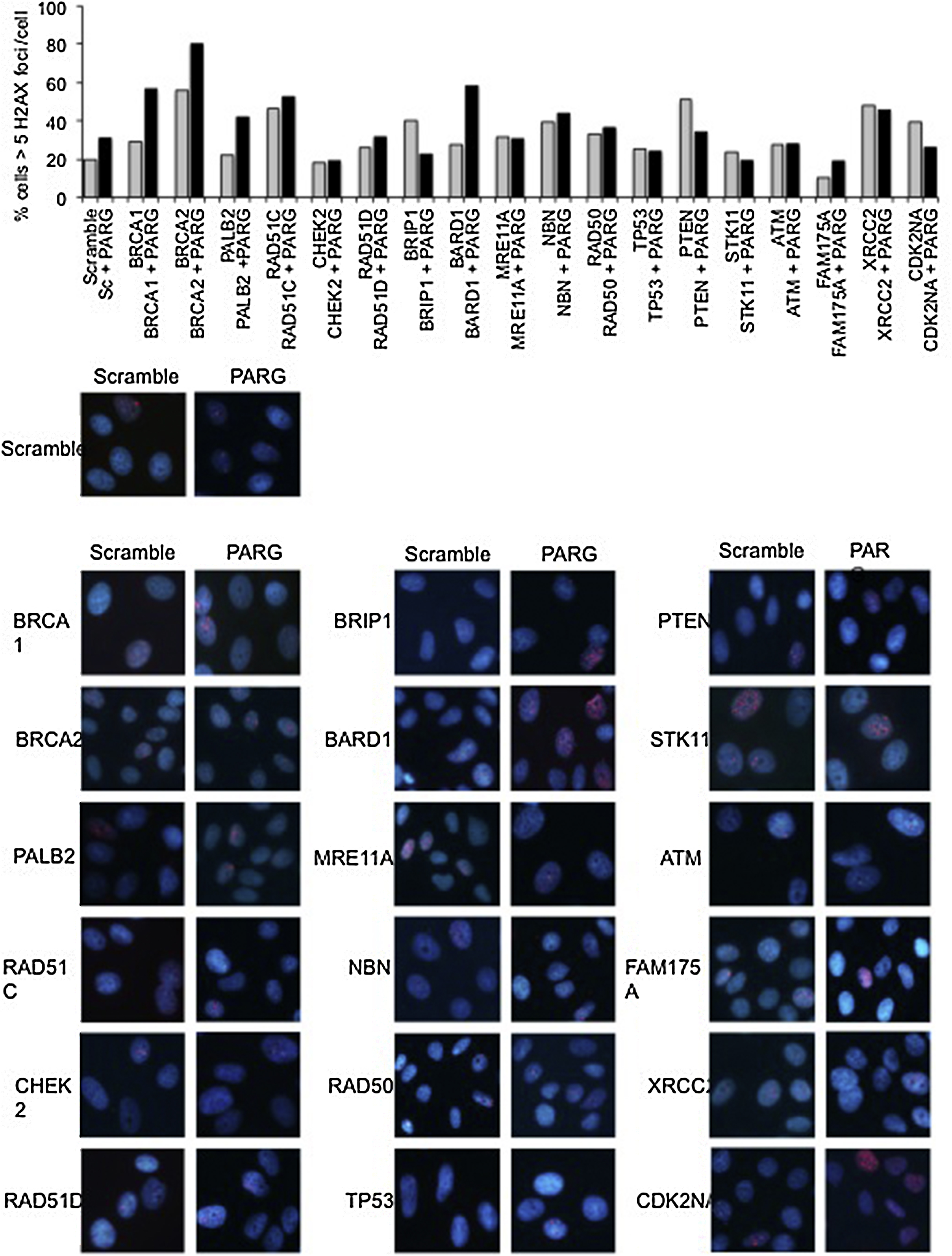

Supplement: Supplementary file 4 [file mmc4.jpg]

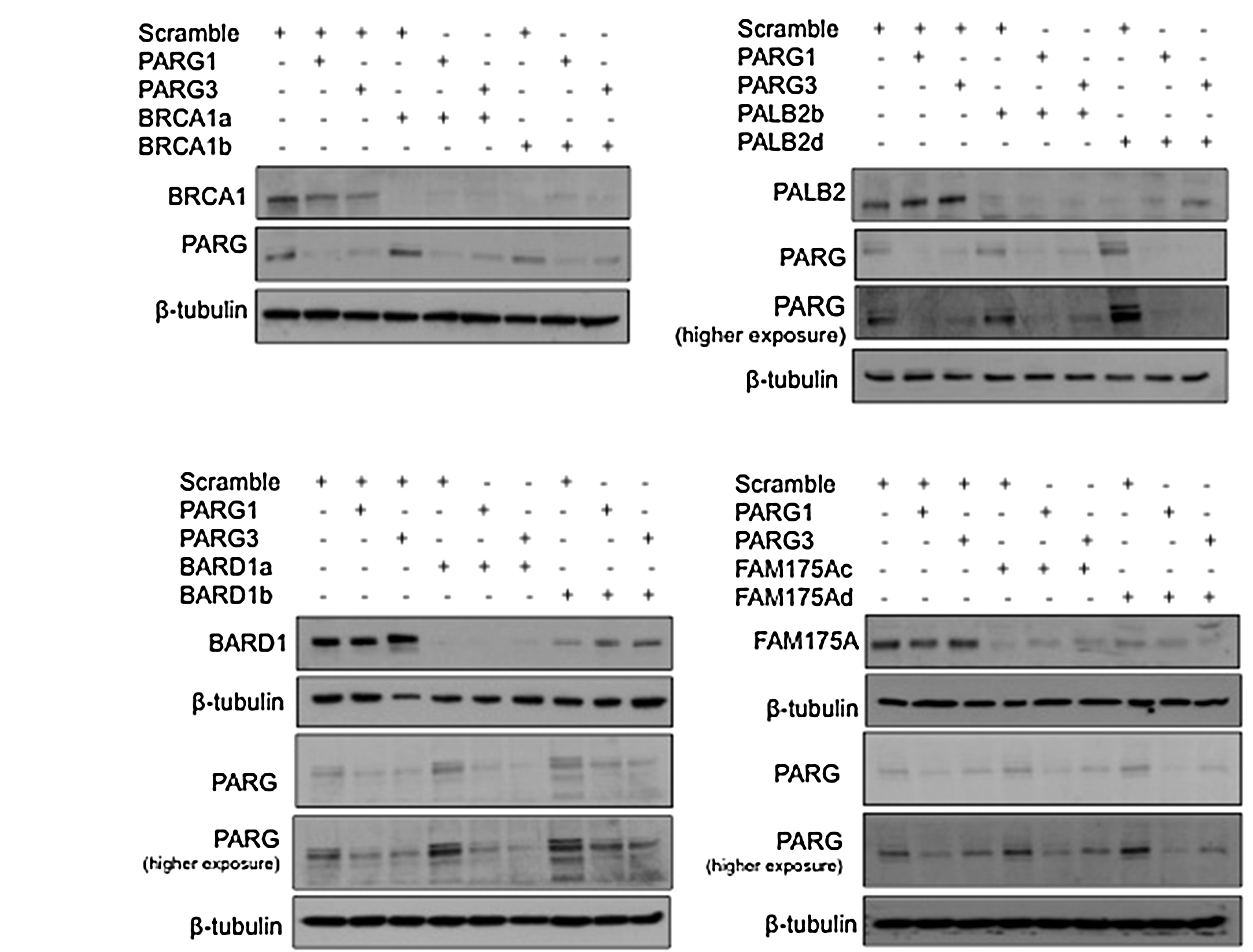

Supplement: Supplementary file 5 [file mmc5.jpg]

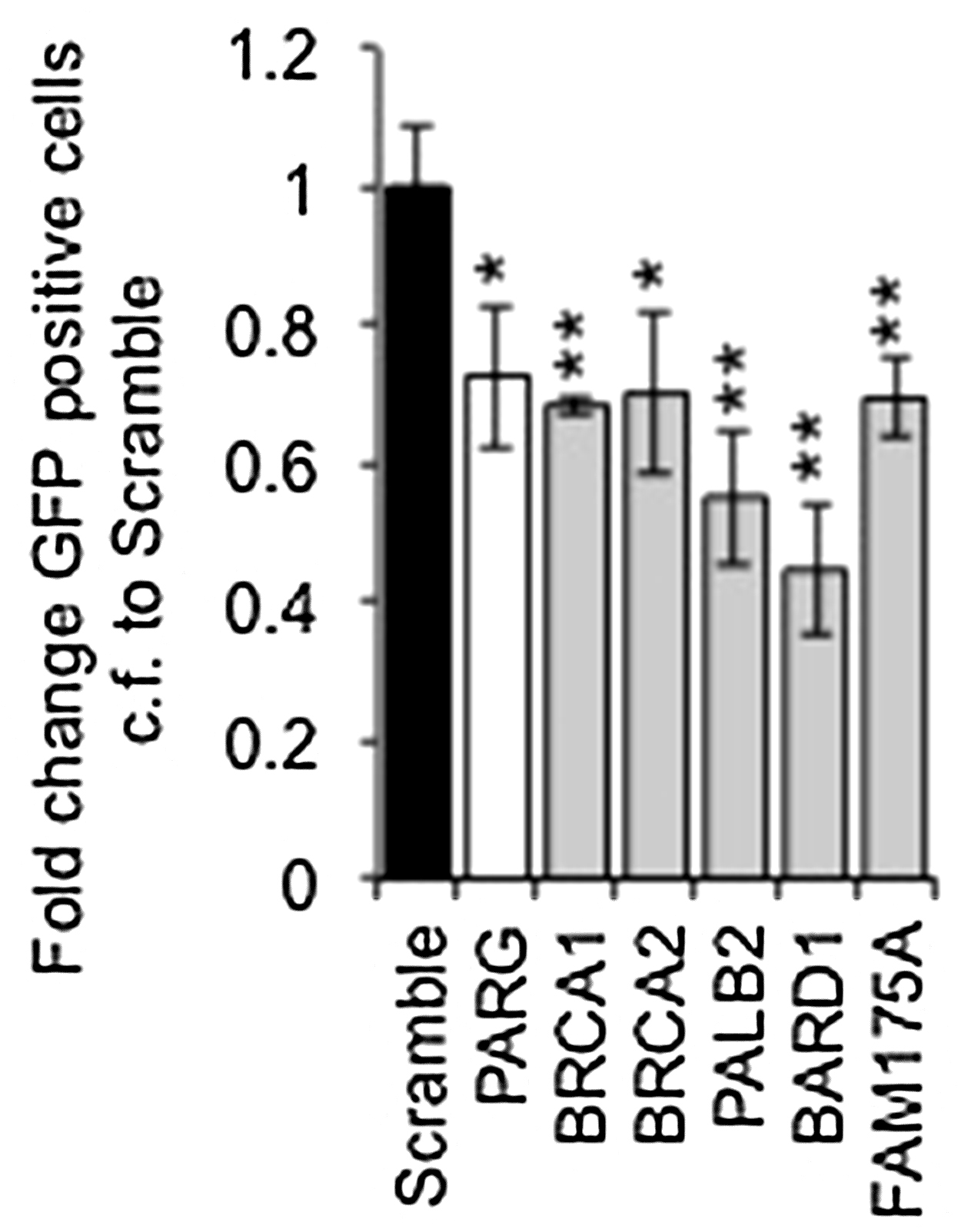

Supplement: Supplementary file 6 [file mmc6.jpg]
